# Exploring the Landscape of Adult Acute Poisoning in Saudi Arabia: A Comprehensive Narrative Review

**DOI:** 10.7759/cureus.66842

**Published:** 2024-08-14

**Authors:** Mohammed D Al Shubbar, Loay M Bojabara, Abdulaziz A Al Qunais, Ammar S Huldar, Saud Alamro, Mohammed H Alameer, Faris H Alameer

**Affiliations:** 1 College of Medicine, Imam Abdulrahman Bin Faisal University, Dammam, SAU; 2 ‏College of Medicine, ‏Imam Abdulrahman Bin Faisal University, Dammam, SAU; 3 Intensive Care Unit, King Fahad Specialist Hospital, Dammam, SAU

**Keywords:** adults, epidemiology, toxicity, saudi arabia, poisoning

## Abstract

This narrative review meticulously examines the intricate landscape of poisoning incidents within Saudi Arabia, delineating the prevalence and characteristics across three main categories: pharmaceutical, illicit, and chemical substances. Pharmaceutical agents, particularly analgesics and antipsychotics, are identified as leading causes of acute adult poisoning, highlighting the paramount role of their widespread accessibility and the potential risks associated with over-the-counter availability. The review underscores the alarming prevalence of over-the-counter analgesics, such as paracetamol and nonsteroidal anti-inflammatory drugs (NSAIDs), as the most frequent culprits in poisoning cases, with a significant correlation observed between analgesic poisoning and gender, notably affecting the female population. Additionally, the study delves into the burgeoning issue of illicit drug use, with opioids and amphetamines emerging as substantial contributors to the poisoning landscape, reflecting a broader global trend of increasing substance misuse and its associated health risks. Chemical poisoning, encompassing a range of substances from household cleaners to industrial chemicals, presents another critical area of concern, with specific emphasis on the dangers posed by antiseptics, detergents, and organophosphates. The review identifies a pressing need for targeted public health interventions and policy reforms aimed at mitigating the risks associated with these diverse types of poisoning. By offering a comprehensive overview of the poisoning epidemiology in Saudi Arabia, the study contributes valuable insights into the multifaceted nature of this public health challenge, advocating for enhanced regulatory measures, public awareness campaigns, and improved access to mental health services to address the underlying factors contributing to poisoning incidents.

## Introduction and background

A poison is a substance that, upon exposure through ingestion, inhalation, or dermal contact, induces harm or injury to biological systems, thereby jeopardizing an individual's health and survival [1]. Such substances are recognized as significant public health issues, persisting even within advanced nations [2-4]. The categorization of poisoning may be delineated according to its type of poison, intentionality, and chronicity.

Intentionality emerges as a pivotal consideration in the analysis of poisoning incidents. It delineates scenarios wherein the exposure to toxic substances is deliberate, such as in cases of self-inflicted harm, contrasted with unintentional instances arising from accidental contact and, under dire circumstances, acts of homicide. Evidence suggests that instances of intentional poisoning often present with greater severity and elevated risk to life when compared to accidental exposures [5]. Furthermore, comparative research indicates geographical disparities in the prevalence of suicidal poisoning. According to a specific investigation, this mode of self-harm is notably prevalent within Scandinavian nations and the United Kingdom, serving as a frequent method of poisoning. Conversely, such occurrences are reported to be infrequent within regions of Eastern Europe, Central and South America, and Asia [6].

Another essential aspect to be considered in toxicology is chronicity, which serves as a critical axis, differentiating poisoning incidents into acute or chronic categories based on their temporal progression and impact. Acute poisoning is described by the immediate and harmful health consequences following singular or multiple toxic exposures over a brief span [7]. This phenomenon transcends national boundaries, presenting a worldwide challenge. A case in point is the year 2008, during which acute poisoning ascended to be the predominant cause of mortality attributed to injuries within the United States [6]. This statistic underscores acute poisoning as a significant public health challenge, meriting global attention and rigorous preventive strategies.

A critical observation to be highlighted is that the poisoning patterns, including the substances involved, vary between pediatric and adult populations. This variation can be vividly illustrated by an empirical study conducted in the United States, which examined the incidence of poisoning-related fatalities stemming from drugs and opioid overdoses over the consecutive years of 2017 and 2018. The study's outcomes indeed demonstrated that, in both years under review, a staggering 99.99% of the deceased were individuals aged 15 years and older [8].

Another pivotal framework for categorizing poisoning incidents involves categorizing poisoning based on the types of toxic agents implicated. This classification encompasses a broad spectrum of substances, including pesticides, medicinal agents and pharmaceuticals, chemicals, illicit drugs, and industrial compounds, among other diverse types [9]. The elucidation of these categories is not merely *taxonomical* but serves a critical function in identifying potential risk factors unique to each type of poisoning. Recognizing these risk factors is instrumental in the formulation and execution of regulatory and policy measures aimed at mitigating the incidence of such adverse outcomes.

Saudi Arabia, a vast nation with a populace nearing 32 million distributed across its 13 regions, grapples with the public health challenge of poisoning [10]. Despite several robust epidemiological investigations within specific regions and cities of the kingdom [11-17], more comprehensive epidemiological data is needed at the national level. This narrative review endeavors to construct an overarching analysis of the predominant methods of acute poisoning among adults within Saudi Arabia, examining the forensic dimensions, including intentionality and mortality rates. Additionally, it aims to evaluate demographic factors, qualitatively, and their influence on poisoning trends. A further objective is to shed light on the regional variances in poisoning patterns throughout the country. This investigation seeks not only to bridge the existing knowledge gap but also to furnish insights that could inform targeted public health strategies and policy interventions tailored to mitigate the impact of poisoning within the Saudi Arabian context.

## Review

Pharmaceutical drugs

Pharmaceutical substances, mainly attributable to their widespread accessibility, are potentially the predominant means of poisoning within Saudi Arabia. For example, an extensive retrospective analysis conducted in Qassim Province - a region boasting a population of over one million - revealed that pharmaceuticals, alongside chemicals, constituted the principal agents of acute adult poisoning in the year 2017 [11].

Analgesics

A diverse array of pharmaceuticals is implicated in cases of poisoning. Yet, it is analgesics, especially paracetamol and NSAIDs, that constitute most acute adult poisoning incidents in Saudi Arabia. In every single study conducted that examined pharmaceuticals as a source of poisoning within the country, analgesics emerged as the most frequently identified category. This pattern is consistent across regions, including Qassim, Makkah, Jeddah, the Eastern Province, Al-Baha, Riyadh, and Najran, suggesting a nationwide trend. The uniformity observed across these regions likely stems from the easy accessibility of these medications, many of which are available over the counter, and their affordability. The association between analgesic poisoning and gender is also notable, with a significant link to the female gender identified in some analyses, including studies from the Eastern and Western Provinces, Al-Baha, and Riyadh [12-15]. Paracetamol recognized globally for its association with suicidal attempts, shows a similar trend in Saudi Arabia. A focused study in the Eastern Province on adult patients who overdosed on paracetamol found that approximately 80% of these instances were suicide attempts [14]. Additionally, comparative research in the Western Province indicated that painkillers are more frequently associated with intentional poisonings than other pharmaceutical drugs [12].

These observations compel a broader examination of the pharmacological landscape in Saudi Arabia. While analgesics represent a significant portion of acute poisoning cases, the exploration of other pharmaceutical drugs is essential for a holistic understanding of the poisoning phenomenon.

Antipsychotics

It is crucial to extend our focus to another significant category of pharmacological agents implicated in such incidents: antipsychotic drugs. These medications, pivotal in managing psychiatric conditions, have emerged as common culprits in acute poisoning cases alongside analgesics. In the year 2017, Qassim province reported that approximately 12% of all adult acute poisoning incidents were attributable to antipsychotics, making it the second most frequent cause of drug poisoning in the region for that year [11]. This finding is echoed in a comprehensive surveillance report spanning from 1999 to 2003, also in Qassim, which highlighted that drugs affecting the central nervous system (CNS)-including antipsychotics-ranked alongside analgesics as the leading causes of drug-induced poisoning [16]. The prominence of antipsychotics in acute poisoning scenarios extends beyond Qassim. For instance, a study conducted in Jeddah between 2011 and 2016 identified antipsychotics as the second most prevalent cause of drug poisoning [12]. However, it's noteworthy that other regions, such as another study in Jeddah, along with Najran and Al-Baha, reported lesser involvement of antipsychotics in acute poisoning cases [13,17,18]. The reasons behind the varying prevalence of antipsychotic-induced poisonings between areas like Qassim and Jeddah, compared to others, remain elusive, underscoring a gap in our understanding that warrants further investigation.

As we continue to explore the spectrum of substances contributing to acute poisoning within Saudi Arabia, the variable impact of antipsychotics across different regions highlights the complexity of this public health issue. It emphasizes the need for region-specific studies to uncover the underlying factors contributing to these trends, ultimately guiding targeted prevention and intervention strategies.

Anticonvulsant medications

Continuing the exploration of pharmacological agents implicated in acute poisoning incidents within Saudi Arabia, the role of anticonvulsant medications warrants particular attention. These drugs, essential for managing seizure disorders, have been identified as a notable cause of acute poisoning among adults in various regions of the country.

In an illustrative study conducted at King Khalid National Guard Hospital in Jeddah from January 2008 to December 2012, anticonvulsant medications accounted for approximately 17.2% of all acute poisoning cases, positioning them just behind analgesics in terms of prevalence [17]. While the specificity of the study population - military employees - may introduces limitations to generalizability, corroborative evidence from Al-Baha reinforces the prominence of anticonvulsants, where they emerged as the third most common cause of acute drug-related poisonings [13]. Further emphasizing the significance of this issue, research from a university hospital in the Eastern Province reported a substantial proportion of overdose toxicity cases attributable to anticonvulsant drugs, specifically phenytoin, valproic acid, and carbamazepine [19]. Conversely, other studies present a contrasting picture; for example, in Najran, anticonvulsants were implicated in only about 2.4% of acute poisoning instances [18], and a similar trend of minimal involvement was observed in Riyadh [15,20]. The apparent regional disparities in the incidence of anticonvulsant medication poisoning underscore a complex interplay of factors that may influence these patterns. While anticonvulsants significantly contribute to acute toxicity in certain areas, their relatively minor role in others prompts questions about the underlying reasons for such variability. This disparity suggests a need for more nuanced investigations to elucidate the mechanisms driving these trends, potentially guiding more effective preventative measures and therapeutic interventions tailored to regional specificities.

Other CNS-affecting drugs

Building upon the discussion of specific pharmaceutical agents such as analgesics, antipsychotics, and anticonvulsants, it becomes clear that drugs impacting the CNS stand out as significant contributors to acute poisoning cases. This broad category encompasses not only the previously mentioned drug classes but also extends to benzodiazepines, antidepressants, and antihistamines. The literature consistently shows that these CNS-affecting substances are commonly implicated in poisoning incidents, with a notable proportion being associated with intentional misuse [13,16,17,21].

Miscellaneous

The issue of acute poisoning, however, is not confined to CNS-active drugs alone. The scope of substances involved in such medical emergencies is broader, including various other pharmacological groups. Among these, antibiotics, anti-hypertensives (blood pressure medications), antidiabetic agents, antiemetics, and asthma therapies are occasionally reported as factors in poisoning cases [11,12,21]. While these categories may not be as frequently cited as the leading causes of poisoning, their presence in the literature underscores the diversity of pharmaceutical agents capable of leading to toxicological emergencies.

Substances

The challenge of illicit drug use is escalating globally, with Saudi Arabia being no exception. This issue engenders many adverse consequences, spanning health, psychological, social, and economic impacts on both the individual and societal levels. Research has consistently shown a rising trend in the consumption of illicit substances within the Saudi population, predominantly among adult males [22]. The repercussions of such drug use are profound, encompassing a range of health complications that can culminate in fatality, particularly in instances of poisoning resulting from the administration of excessively high dosages of these substances [23].

Opioids

Heroin, an exceedingly hazardous opioid, has emerged as a significant concern in Saudi Arabia, with its abuse extensively documented across various studies. Research conducted within addiction treatment centers in the country reveals a staggering range of heroin dependency among Saudi patients, with prevalence rates fluctuating between 6.6% and 83.6% [24]. This wide range underscores the gravity of heroin abuse within the nation's demographic spectrum.

In the Eastern Province, a particular investigation aimed at assessing admissions for drug-related issues disclosed that out of 5,574 admissions, 18 were directly attributable to drug abuse, encompassing opioids, among other substances. Yet, this study did not specify the demographic details, such as the most affected age group or gender [19]. Conversely, a study in Jeddah focusing on heroin-related postmortem analyses conducted by the Jeddah poison control center presented a more detailed demographic insight. It reported that out of the deceased, 97% to 98% were male, predominantly heroin-dependent, with the majority falling within the middle-aged bracket, having a median age of 38 years [25]. These findings not only highlight heroin as a critical factor contributing to substance abuse and related fatalities in Saudi Arabia but also illustrate a pronounced gender disparity among those affected. The high dependency rates observed in addiction treatment facilities, coupled with the demographic specifics gleaned from postmortem data, reflect the urgent need for targeted interventions.

Stimulants

Amphetamine, a stimulant prescribed for certain psychiatric conditions, is also subject to misuse by individuals seeking its energizing effects. A comprehensive review highlighted amphetamine as one of the substances most frequently abused within addiction treatment contexts, with abuse rates ranging from 4% to 70.7%. Notably, the prevalence of amphetamine misuse has seen an uptick over the past decade [26], signaling a growing concern within public health spheres. In a specific study conducted in Najran, researchers explored the incidence of acute intoxication among adults. The findings revealed that, of 852 participants, 120 had experienced intoxication due to amphetamine, making it the second leading cause of intoxication identified in the investigation. Most of these cases were reported in individuals aged 26 to 35 years. However, the study did not specify the gender most affected by amphetamine use, although it was observed that female patients exhibited a slightly higher rate of poisoning across the study's overall sample [18]. This gender-related data point adds a layer of complexity to the issue of amphetamine intoxication, hinting at nuanced differences in substance abuse patterns among different demographic groups. The absence of region-wide studies on amphetamine poisoning in Saudi Arabia leaves a significant gap in our understanding of the geographical distribution of stimulant misuse. Without comprehensive data from various regions, it is challenging to ascertain whether the southern region, as represented by the Najran study, is more affected by stimulant poisoning than other areas.

Alcohol

Alcohol abuse emerges as a significant concern within the realm of substance misuse in Saudi Arabia, as illuminated by the review article that also discussed stimulant abuse. This analysis revealed that between 9% and 70.3% of individuals seeking help in addiction treatment settings are struggling with alcohol dependency [26], underscoring the pervasive nature of alcohol abuse across various demographics within the country. In alignment with findings related to stimulant intoxication, the study from Najran that was previously referenced in the context of amphetamine also shed light on alcohol-related intoxications. It documented 90 cases of alcohol poisoning, predominantly among individuals older than 35 years. However, similar to the amphetamine study, this investigation did not delineate the most affected gender for alcohol intoxication precisely, although it was noted that female patients exhibited slightly higher rates of poisoning overall in the study's findings [18].

Further contributing to the understanding of alcohol misuse in Saudi Arabia, a separate investigation conducted in Hail focused on reported instances of alcohol poisoning and consumption at King Khalid Hospital. This study spanned a wide age range of patients, from 19 to 75 years old, revealing a trend of higher alcohol consumption among males. This gender disparity suggests that males are at an elevated risk for experiencing complications from alcohol use, including poisoning [27].

Chemicals

A total of 15 studies were done in the Kingdom of Saudi Arabia mentioning chemical poisoning epidemiology among the population [11,15-21,28-34]. The studies were conducted across Saudi Arabia using different methodologies; two were case series [33,34], and the rest were retrospective reviews of medical records. There was no consensus on adults' age, with some studies considering 15 years and above as adults. The pediatric population was excluded from the analysis. A total of 1,385 cases of chemical poisoning were collected from 15 studies. Chemicals included in the studies are disinfectants and antiseptics, detergents, insecticides, pesticides, hydrocarbons, and methanol.

Antiseptics and Disinfectants

Antiseptics and disinfectants are used on surfaces to kill microorganisms; despite no evidence of protection against infections, they are widely used in almost all houses in Saudi Arabia, and they pose a considerable risk of poisoning [35].

A study done in Al-Qassim concluded that antiseptic and disinfectant were responsible for 93 cases of poisoning [29]. Another study in Jeddah reported 105 cases and was the second most common cause after detergents [28]. It poses a higher risk due to accessible acceptability. In most Saudi houses, it is usually kept in the kitchen, which also is a risk for cross-contamination with food.

Detergents

Detergents, integral to laundry practices with standard types, including Clorox and laundry pods, represent a significant source of chemical poisoning within Saudi Arabia. In a study conducted in Jeddah, detergent-related incidents were identified as the leading cause of chemical poisoning among adults, accounting for 115 cases [28]. Notably, this study did not differentiate between children and adults in its analysis of gender and intentionality. However, it was reported that detergents were involved in 56.3% of cases where ingestion was intentional across all chemical poisoning incidents. This trend may be attributed to the widespread availability and affordability of detergents, making them more accessible than other chemical substances and present in nearly all households.

Further research in Al Majmaah highlighted the issue of bleach poisoning, with five reported cases [21], while a study in Al Baha documented 16 instances of Clorox poisoning [13]. These figures suggest that detergent poisoning rates are comparatively higher, though there is some variability in reporting, with specific studies categorizing these incidents under the broader term *household products*. This category encompasses antiseptics, detergents, and insecticides, among others, leading to a need for more consensus in the terminology used across different studies.

Organophosphates

Organophosphate compounds, widely utilized in pesticides and insecticides for crop protection against infestations, present a notable chemical hazard [36]. The body of literature on organophosphate poisoning from Saudi Arabia reveals a degree of ambiguity, with specific studies broadly referencing pesticides or insecticides without specifying whether they are organophosphate-based. Nonetheless, specific research, such as a study from Al Khobar, provides clarity, documenting 50 cases of organophosphate poisoning, 39 of which involved adults [31]. An analysis of these cases indicated that males constituted 68% of the incidents, with 58% being accidental, 18% intentional, and the remainder unspecified. Furthermore, ingestion emerged as the most prevalent route of exposure, followed by inhalation, while dermal contact was the least common. In Al Qassim, another investigation highlighted insecticides as the leading cause of poisoning, accounting for 157 cases. While not explicitly stated, the study did not confirm all cases were due to organophosphate poisoning. It also suggested a rising trend in such incidents, potentially linked to the region's agricultural and industrial expansion [29].

Aluminum phosphide, known for its application as an insecticide, is characterized by its high toxicity and consequentially elevated mortality rates. Highlighting its profound impact, a comprehensive nationwide study from 2006 to 2017 reported 26 documented cases of aluminum phosphide poisoning [32]. This figure, while seemingly modest, underscores the lethal potential of this chemical when exposures occur, emphasizing the critical importance of stringent handling, usage, and regulatory measures to mitigate the risks associated with its use.

Methanol

Methanol poisoning represents a critical public health concern, characterized by its potential to induce acute multi-organ failure and irreversible vision loss. As a substance widely used in the manufacture of airplane fuels and perfumes, methanol's toxicity is profound, with ingestion of as little as 10 mL capable of resulting in fatal outcomes. In Saudi Arabia, the majority of methanol poisoning cases arise from the consumption of counterfeit alcohol or through suicidal attempts involving colognes [33]. Such incidents often occur in clusters, leading to outbreaks, mainly when groups consume counterfeit alcohol. The prohibition of alcohol within the kingdom prompts some individuals to resort to the production of homemade alcohol, inadvertently increasing the risk of methanol toxicity.

Research on methanol poisoning within Saudi Arabia, however, still needs to be explored, with only three studies addressing the issue. Two were case series, collectively documenting 17 cases [33,34]. Additionally, another study conducted in Al Baha reported 23 instances of methanol poisoning [13]. This lack of research underscores a pressing need for more extensive investigation into methanol poisoning in the region. Enhanced research efforts are essential to understanding the scope of this problem better, developing effective prevention strategies, and implementing measures to mitigate the risks associated with methanol exposure. Given the severe consequences of methanol poisoning, prioritizing this study area is crucial for safeguarding public health and preventing future outbreaks.

Fuels

While hydrocarbons such as kerosene, benzene, and gasoline are not frequently implicated in poisoning cases, their impact is nonetheless significant within Saudi Arabia. From the available literature, 48 cases have been reported across two studies, indicating that incidents involving these substances are reported more frequently than those involving methanol [13,29]. One of these studies notably found that females are more susceptible to fuel poisoning compared to males, suggesting gender differences in exposure or vulnerability.

Despite identifying these cases, the studies in question needed a more detailed examination of fuel toxicity, particularly regarding the mode of exposure, the intentionality behind the poisoning incidents, and the subsequent outcomes for the affected individuals.

Animal envenomation

Snakebites represent a significant public health issue in Saudi Arabia, particularly affecting those engaged in agricultural activities within rural communities. The incidence of snakebites is associated with heightened rates of mortality and morbidity, underscoring the urgent need for effective preventive measures and treatment options tailored to the needs of individuals most at risk, such as workers in the farming and plantation sectors [37]. Similarly, scorpion envenomation poses a substantial threat in various global regions, including Saudi Arabia, where knowledge about its prevalence and impact remains limited [38]. The challenges presented by snakebites and scorpion envenomation highlight the necessity for comprehensive research efforts within Saudi Arabia. Such studies would provide valuable insights into the epidemiology of these incidents, raising awareness among vulnerable populations.

Snakebites

A comprehensive nationwide study encompassing all hospitals and health centers from 2015 to 2018 revealed a total of 14,697 snake bite incidents, averaging 3,674.25 cases annually. The data indicated that 2016 witnessed the peak in snakebite occurrences, with August registering the highest incidence rates. According to the findings of this study, male victims predominated, constituting 80% of the cases, leaving the remaining 20% as female victims [37]. This substantial number of snakebite cases underscores the critical need for heightened awareness, preventive strategies, and accessible medical care, especially in high-risk areas. The gender disparity in snakebite victims suggests potential differences in occupational or behavioral exposure, warranting targeted interventions. Moreover, the seasonal peak in August may reflect ecological or agricultural patterns influencing snake activity, pointing to specific periods when preventive measures should be intensified.

Scorpion Stings

Scorpion envenomation poses a significant threat across various global regions. Nationwide research estimates an annual occurrence of approximately 14,500 cases of scorpion stings across different Saudi areas, underscoring the magnitude of this issue [39]. The data indicates a seasonal pattern, with the highest incidence rates occurring during summer, reflecting the scorpions' increased activity in warmer temperatures.

A detailed study from 1999 to 2003 documented 6,465 scorpion sting incidents in Qassim province alone, remarkably without any fatalities reported [40]. This may suggest both an effective local medical response to scorpion envenomation and that scorpion species habituating in Saudi Arabia might be a minor threat as the species seen in different countries. Despite the absence of death, this does not diminish the potential severity of such incidents. In Al-Jouf, another region of Saudi Arabia, 1,449 scorpion sting cases were recorded over two years (2005-2006), with most of the victims being male. Additionally, the study observed a higher frequency of stings during daylight hours than at nighttime [41].

The patterns of poisoning in Saudi Arabia are complex and multifaceted. Table 1 and Table 2 provide a comprehensive overview of the epidemiology of poisoning across various regions and categories. With a clear understanding of these patterns, it is crucial to examine the associated mortality rates and the intentionality behind these incidents to develop effective public health interventions.

**Table 1:** Demographic characteristics and common causes of poisoning in each included article.

Author	Region	Number of participants	Most common cause of poisoning	Most common affected age	Dominant gender
Alzahrani et al. (2017) [12]	Jeddah	1474	Analgesics	15-24 years old (17.5%) and older than 24 years (21.9%)	Female (60%)
Alzahrani et al. (2017) [28]	Jeddah	994	Detergent	15-24 years old (34.6%)	Male (54.6%)
Alnasser et al. (2022) [29]	Al Qassim	281	Disinfectant	Not mentioned	Males 52.2%
Beigh et al. (2023) [13]	Baha Province	622	Medications, particularly analgesics, and antipsychotic	The most vulnerable group was those aged 1 to 20 years.	Males
Alnasser et al. (2020) [11]	Al Qassim	381	Analgesics	The mean age is not mentioned, but the data involves multiple age groups.	For drugs Female
					For food Female
					For chemicals Male
Alghamdy et al. (2015) [19]	University hospital in the Kingdom of Saudi Arabia (did not mention the location exactly)	253	Overdose and side effects of drugs	Not mentioned	Not mentioned
Hegazy et al. (2012) [30]	Makkah	200	Acetaminophen	Adults (60.6%)	Female
Almansori et al. (2015) [14]	Khobar	86	Acetaminophen	24.4 +/- 6.6	Female (72%)
Bakhaidar et al. (2015) [17]	Jeddah	128	Analgesics	12-35 years old (33.3%) and >35 years old (22.5%)	Females 54.3%
Alghafees et al. (2022) [20]	Riyadh	1,505	Acetaminophen	492 adults	Males
Wahba et al. (2021) [18]	Najran	852	Drug intoxication most commonly acetaminophen and amphetamine	15-25 years old (53.9%), 26-35 years old (25.5%), and >35 years old (20.6%)	Female (53.5%)
Bintalib et al. (2022) [15]	Riyadh	108	Acetaminophen	Mean age is 33 years old	Males (65.7%)
Al-Asmari et al. (2023) [25]	Jeddah	97	Heroin	Middle age	Male
Alhaidan et al. (2022) [27]	Hail	550	Alcohol	Mean age of group 1 was 28 +/- 9 and mean age of group 2 was 32+/-10	Males
Abd-Elhaleem and Al Muqhem (2014) [21]	Al Majmaah	591	Animal envenomation (62%) of which scorpion stings were the most common.	79.4% >12 years old	Males (73.6%)
Alghamdy et al. (2015) [19]	University hospital in the Kingdom of Saudi Arabia (did not mention the location exactly)	253	Overdose and side effects of drugs	Not mentioned	Not mentioned
Hegazy and Almalki (2012) [30]	Makkah	200	Acetaminophen	Adults (60.6%)	Female

**Table 2:** Poisoning incidents in four major regions in Saudi Arabia (eastern, western, middle, and southern) categorized according to the type of poisoning (medicine, substances, chemical agents, and animal envenomation).

Region/Substance	No. of studies	No. of patients	% of all poisoning
Western region			
Total	9	2,824	100%
Medicine		654	23%
Substances		167	6%
Chemical Agents		383	14%
Animal envenomation		1,509	53%
Not specified		111	4%
Middle region			
Total	8	8,027	100%
Medicine		499	6%
Substances		270	3%
Chemical agents		688	9%
Animal envenomation		6,514	81%
Not specified		56	1%
Eastern region			
Total	3	229	100%
Medicine		162	71%
Substances		18	8%
Chemical Agents		49	21%
Animal envenomation		0	0%
Not specified		0	0%
Southern region			
Total	1	852	100%
Medicine		513	60%
Substances		90	11%
Chemical agents		249	29%
Animal envenomation		0	0%
Not specified		0	0%
Nationwide			
Total	2	11,769	100%
Medicine		0	0%
Substances		0	0%
Chemical agents		26	0.22%
Animal envenomation		11,743	99.78%
Not specified		0	0%

Mortality and intentionality 

The ramifications of poison-related incidents are profound, exerting substantial impacts on global and local health landscapes by significantly contributing to morbidity and mortality while also imposing a considerable financial strain on healthcare systems. Mortality arising from poisoning can manifest through both deliberate and accidental circumstances. Notably, suicide ranked as the fourth leading cause of death among individuals aged 15-29 years worldwide in 2019, with pesticide self-poisoning constituting 20% of these suicides, as highlighted by the World Health Organization (WHO) [42].

WHO further reports that the mortality rate due to unintentional poisoning stood at 0.82% in 2019, though it did not provide specific figures for intentional poisoning [43]. The exploration of suicide methodologies within the Saudi Arabian context reveals a lack of data, particularly concerning self-poisoning. A narrative review conducted in 2021 examining suicide studies within the nation identified hanging, firearms, and jumping from heights as the predominant methods, suggesting a lack of significant findings related to self-poisoning [44].

This section comprehensively analyzes the mortality and intentionality rates associated with poisoning, drawing upon data from 16 distinct studies. These studies cover a range of poisoning types, revealing critical insights into the patterns and outcomes of poisoning incidents across different regions of Saudi Arabia.

Mixed Poisoning Studies

The analysis of poisoning incidents across Saudi Arabia, as derived from eight mixed studies, provides a nuanced understanding of the prevalence, mortality, and intentionality associated with various types of poisonings across different regions (Table 3). Collectively, these studies encompass a broad spectrum of poisoning incidents, offering insights into regional trends and specific characteristics of poisoning cases [11,13,15-18,20,30].

**Table 3:** Summary of mortality rate and intentionality in included articles. *For alcohol abuse patients.

Type of poisoning	Authors	Intentional (Suicidal and homicidal)	Mortality
Medical poisoning	Alzahrani et al. (2017) [12]	28%	0%
	Alghamdy et al. (2015) [19]	Not mentioned	4%
	Almansori et al. (2015) [14]	80%	0%
Substance abuse	Al-Asmari et al. (2023) [25]	7% suicidal; 5% homicidal	Not mentioned
	Alhaidan et al. (2022) [27]	Not mentioned	0.4%*
Chemical agents	Alqurashi et al. (2017) [33]	15.90%	1.10%
	Alnasser et al. (2022) [29]	22.60%	2.60%
	Alnasser et al. (2018) [32]	0%	32%
	Jumaan et al. (2015) [31]	18%	4%
Studies with variable types of poisoning	Bakhaidaret et al. (2014) [17]	43.05%	0%
	Hegazy and Almalki (2012) [30]	51% (Suicidal)	1%
	Wahba et al. (2021) [18]	35%	1.20%
	Alnasser et al. (2020) [11]	12.22% for drug poisoning; 3.7% for chemical poisoning	Not mentioned
	Bintalib et al. (2022) [15]	Not mentioned	2%
	Beigh et al. (2023) [13]	3.7	0.2
	Alghafees et al. (2022) [20]	11.2%	0.80%
	Moazzam et al. (2009) [16]	27.00%	2.20%

Regional distribution: The studies were geographically distributed, with three conducted in the western regions, four in the central areas, and one in the southern region of Saudi Arabia [11,13,15-18,20,30]. This distribution allowed for comparative analysis across different parts of the country, reflecting the variable nature of poisoning incidents.

Najran study (Southern region): The study conducted in Najran, notable for its extensive sample size, reported a mortality rate of 1.2%, the third highest, and the highest percentage of intentional poisoning at 35.3% [24]. These figures highlight a significant concern regarding the prevalence of intentional poisonings in this region [18].

Al-Qassim study (Central region): In contrast, Al-Qassim reported the overall highest mortality rate of 2.2% despite having a smaller sample size than Najran. Furthermore, it was observed that pesticides were the predominant cause of death in 24.7% of fatal poisoning cases [16], underscoring the critical impact of pesticide exposure in the central region.

Makkah study (Western region): Unique among the reviewed studies, the research conducted in Makkah provided additional insights into the nature of intentional poisonings, revealing that 25% of such cases involved individuals with psychiatric issues [30]. This finding emphasizes the intersection between mental health and the risk of intentional poisoning.

Age group analysis: Another study from the western region identified that the demographic group most susceptible to intentional poisoning was individuals aged between 12 and 35 years [17]. This age-specific trend indicates a potentially vulnerable population group that may require targeted prevention and intervention efforts.

These studies collectively underscore the complex and multifaceted nature of poisoning incidents across Saudi Arabia. The varied mortality rates, the significant proportion of intentional poisonings, and the notable impact of pesticides as a cause of fatal poisonings reveal critical areas for public health focus. Additionally, the association between intentional poisonings and psychiatric conditions highlights the need for integrated mental health services as part of the poisoning prevention and response strategies.

Specific Poisoning Studies

Specifically, four studies were done in the west and central regions for chemical agents (Table 3) [29,31-33]. Out of these four studies, the one done in Al-Qassim - central region - showed the highest rate of intentional poisoning by chemical agents, a staggering 22.6% mortality rate [29]. On the other hand, papers specifically addressing pharmaceutical poisonings encompassed three studies [12,14,19]. The peak mortality rate identified within these was 4%, and the survey dedicated to paracetamol overdoses noted an intentionality rate as high as 80%, which was the highest among all other studies (Table 3) [14,19]. A study done to investigate substance abuse poisoning stated that 5% of intentional poisoning was homicidal [25].

## Conclusions

In conclusion, the multifaceted issue of poisoning presents a significant public health challenge across Saudi Arabia, with substantial implications for morbidity, mortality, and the healthcare system. The exploration of poison-related incidents reveals a complex landscape characterized by a variety of substances implicated in both intentional and unintentional poisonings. The prevalence of pharmaceutical compounds, particularly over-the-counter analgesics, as the most common cause of poisoning underscores the critical role of accessibility and public awareness in the incidence of these events. The examination of mortality and intentionality rates across different regions and substances, including chemical agents, drugs, and specific compounds such as heroin, aluminum phosphide, and paracetamol, highlights the diverse nature of poisoning incidents. Notably, the high intentionality rate associated with paracetamol poisoning emphasizes the need for targeted interventions to address potential underlying issues of mental health and substance misuse.

Additionally, poison control centers play a crucial role in the monitoring and management of toxicological exposures. These centers provide immediate, expert guidance on the treatment of poisoning cases and are vital in collecting data on poisoning trends, which can inform public health strategies. By enhancing the capacity and reach of poison control centers, Saudi Arabia can improve its response to poisoning incidents, thereby reducing morbidity and mortality associated with toxicological exposures.

Furthermore, the studies reviewed indicate significant regional variations in poisoning patterns, with differences in the prevalence of specific poisonings and the most affected demographic groups. These variations underscore the need for region-specific public health strategies and policies to address the unique challenges posed by poisoning incidents. The insights garnered from this comprehensive review illuminate the importance of enhancing public awareness, improving access to mental health services, and implementing stricter regulations on the availability of potentially harmful substances. By addressing the multifaceted aspects of poisoning - from prevention and education to treatment and policy interventions - Saudi Arabia can make significant strides in reducing the impact of poisoning on its population, thereby safeguarding public health and reducing the burden on the healthcare system.

## References

[1] Thomas WF, John HD, Willium RH (2007). Stedman's Medical Dictionary. 28th ed. New York: Lippincott William and Wilkins. Stedman's Medical Dictionary, p. 2004, 28th ed.

[2] Wang L, Wu Y, Yin P (2018). Poisoning deaths in China, 2006-2016. Bull World Health Organ.

[3] Franklin RL, Rodgers GB (2008). Unintentional child poisonings treated in United States hospital emergency departments: national estimates of incident cases, population-based poisoning rates, and product involvement. Pediatrics.

[4] Klein-Schwartz W, Smith GS (1997). Agricultural and horticultural chemical poisonings: mortality and morbidity in the United States. Ann Emerg Med.

[5] Lauterbach M, Solak E, Kaes J, Wiechelt J, Von Mach MA, Weilemann LS (2005). Epidemiology of hydrogen phosphide exposures in humans reported to the poison center in Mainz, Germany, 1983-2003. Clin Toxicol (Phila).

[6] Ajdacic-Gross V, Weiss MG, Ring M, Hepp U, Bopp M, Gutzwiller F, Rössler W (2008). Methods of suicide: international suicide patterns derived from the WHO mortality database. Bull World Health Organ.

[7] (2019). The IUPAC Compendium of Chemical Terminology: The Gold Book. International Union of Pure and Applied Chemistry.

[8] Wilson N, Kariisa M, Seth P, Smith H 4th, Davis NL (2020). Drug and Opioid-Involved Overdose Deaths - United States, 2017-2018. MMWR Morb Mortal Wkly Rep.

[9] Mittal C, Singh S, Kumar-M P, Varthya SB (2021). Toxicoepidemiology of poisoning exhibited in Indian population from 2010 to 2020: a systematic review and meta-analysis. BMJ Open.

[10] (2023). Population estimates by gender, nationality, and region 2010 - 2022, statistical database, general authority of statistics, Saudi Arabia. General Authority of Statistics. https://www.stats.gov.sa/en/43.

[11] Alnasser S, Hussain SM, Alnughaymishi IM, Alnuqaydan AM (2020). Pattern of food, drug and chemical poisoning in Qassim region, Saudi Arabia from January 2017 to December 2017. Toxicol Rep.

[12] Alzahrani SH, Alqahtani AH, Farahat FM, Elnour MA, Bashawri J (2017). Drug poisoning and associated factors in western Saudi Arabia: a five-year retrospective chart review (2011-2016). Pak J Med Sci.

[13] Beigh S, Mahzari A, Alharbi RA, Al-Ghamdi RA, Alyahyawi HE, Al-Zahrani HA, Al-Jadani S (2023). A retrospective study of epidemiological correlations of food, drug and chemical poisoning in Al-Baha, western Saudi Arabia. Healthcare (Basel).

[14] Almansori MA, Alhammadi HI, Almulhim FA (2015). Paracetamol overdose: analysis of a sample from a tertiary hospital in Eastern. Saudi J Med Med Sci.

[15] Bintalib M, Aljthalin RA, Abdu M (2022). Clinical profile and outcomes of poisonings and drug overdose at King Saud Medical City. Int J Pharmaceut Biomed Sci.

[16] Moazzam M, Al-Saigul AM, Naguib M, Al Alfi MA (2009). Pattern of acute poisoning in Al-Qassim region: a surveillance report from Saudi Arabia, 1999-2003. East Mediterr Health J.

[17] Bakhaidar M, Jan S, Farahat F, Attar A, Alsaywid B, Abuznadah W (2015). Pattern of drug overdose and chemical poisoning among patients attending an emergency department, western Saudi Arabia. J Community Health.

[18] Wahba MA, Alshehri BM, Hefny MM, Al Dagrer RA, Al-Malki SD (2021). Incidence and profile of acute intoxication among adult population in Najran, Saudi Arabia: a retrospective study. Sci Prog.

[19] Alghamdy MS, Randhawa MA, Al-Wahhas MH, Al-Jumaan MA (2015). Admissions for drug-related problems at the Emergency Department of a University Hospital in the Kingdom of Saudi Arabia. J Family Community Med.

[20] Alghafees MA, Abdulmonen A, Eid M (2022). Poisoning-related emergency department visits: the experience of a Saudi high-volume toxicology center. Ann Saudi Med.

[21] Abd-Elhaleem ZA, Al Muqhem BA (2014). Pattern of acute poisoning in Al Majmaah Region, Saudi Arabia. Am J Clin Exp Med.

[22] Saquib N, Rajab AM, Saquib J, AlMazrou A (2020). Substance use disorders in Saudi Arabia: a scoping review. Subst Abuse Treat Prev Policy.

[23] Shukla L, Ghadigaonkar DS, Murthy P (2019). Poisoning with drugs of abuse: identification and management. Indian J Crit Care Med.

[24] Al-Jerani F, Al-Basry E, Aldawood H (2019). Substance abuse among Saudi population. Int J Med Dev Countries.

[25] Al-Asmari AI, Alharbi H, Al-Zahrani AE, Zughaibi TA (2023). Heroin-related fatalities in Jeddah, Saudi Arabia, between 2008 and 2018. Toxics.

[26] Bassiony M (2013). Substance use disorders in Saudi Arabia: review article. J Subst Use.

[27] Alhaidan T, Alzahrani AR, Alamri A (2022). Reported cases of alcohol consumption and poisoning for the years 2015 to 2022 in Hail, Saudi Arabia. Int J Environ Res Public Health.

[28] Alzahrani SH, Ibrahim NK, Elnour MA, Alqahtani AH (2017). Five-year epidemiological trends for chemical poisoning in Jeddah, Saudi Arabia. Ann Saudi Med.

[29] Alnasser SM, Kordi TS, Asiri AA, Gupta DK, Alfadl AA, Hussain AS (2022). Epidemiology of chemical poisoning among adults in Qassim region: an eight-year study. Toxics.

[30] Hegazy R, Almalki WH (2012). Pattern of acute poisoning in Makkah region Saudi Arabia, 2009 - 2011. Egyptian J Commun Med.

[31] Jumaan MA Al, Shahrani MS Al, Wahhas MH Al (2015). Organophosphate poisoning: a 10-year experience at a tertiary care hospital in the Kingdom of Saudi Arabia. Saudi J Med Med Sci.

[32] Alnasser S, Hussain SM, Kirdi TS, Ahmed A (2018). Aluminum phosphide poisoning in Saudi Arabia over a nine-year period. Ann Saudi Med.

[33] Alqurashi GI, Alqurashi FS, Alhusayni KM (2023). Case reports study on methanol poisoning in King Abdul Aziz specialist hospital, Taif, Saudi Arabia. J Clin Med.

[34] Eskandrani R, Almulhim K, Altamimi A (2022). Methanol poisoning outbreak in Saudi Arabia: a case series. J Med Case Rep.

[35] Olson KR, Smollin CG, Anderson IB (2022). Antiseptics and Disinfectants, 8th ed.

[36] Adeyinka A, Muco E, Regina AC (2024). Organophosphates. https://www.ncbi.nlm.nih.gov/books/NBK499860/.

[37] Al-Sadoon MK, Fahad Albeshr M, Ahamad Paray B, Rahman Al-Mfarij A (2021). Envenomation and the bite rate by venomous snakes in the kingdom of Saudi Arabia over the period (2015-2018). Saudi J Biol Sci.

[38] Al-Sadoon MK, Jarrar BM (2003). Epidemiological study of scorpion stings in Saudi Arabia between 1993 and 1997. J Venom Anim Toxins Incl Trop Dis.

[39] Alhamoud MA, Al Fehaid MS, Alhamoud MA, Alkhalifah AA, Alzoayed MH, Menezes RG (2021). Scorpion stings in Saudi Arabia: an overview. Acta Biomed.

[40] Jahan S, Mohammed Al Saigul A, Abdul Rahim Hamed S (2007). Scorpion stings in Qassim, Saudi Arabia--a 5-year surveillance report. Toxicon.

[41] Jarrar BM, Al-Rowaily MA (2008). Epidemiological aspects of scorpion stings in Al-Jouf Province, Saudi Arabia. Ann Saudi Med.

[42] Suicide Suicide (2023). Suicide. World Health Organization.

[43] (2023). Poison Control and Unintentional Poisoning. https://www.who.int/data/gho/data/themes/topics/indicator-groups/poison-control-and-unintentional-poisoning.

[44] Altaqaq G, Alsamahiji B, Alabkary S (2021). Suicide in Saudi Arabia: a review. Int J Med Dev Countries.

